# People Are Unwilling to Help Others Pursue a Luxury Life: Egocentric or Other-Centric Motivations?

**DOI:** 10.3390/bs16020306

**Published:** 2026-02-22

**Authors:** Jian Hao, Shiqing Li, Weiran Li

**Affiliations:** School of Psychology, Capital Normal University, Beijing 100048, China

**Keywords:** agreeableness, helping, motivation, prosocial behaviour

## Abstract

People are generally willing to help others maintain a basic life, but their willingness to help others pursue a luxury life—and the motivations underlying such decisions—remain unclear. Study 1 examined willingness to help and emotion expectancy in response to demand for necessary versus luxury items, taking helpers’ agreeableness and the controllability of the causes of others’ adversity into account. Study 2 further tested whether helpers’ cost of helping or the utilitarian goals of what others wanted would explain willingness to help and emotion expectancy. A total of 308 university students, acting as potential helpers, were randomly assigned to different helping scenarios. Study 1 found that demand for luxury items reduced both willingness to help and expected happiness, regardless of personality or situational factors. Study 2 showed that among participants low in agreeableness, low utilitarian goals consistently reduced willingness to help, suggesting an other-centric motivation. Among participants high in agreeableness, low utilitarian goals reduced willingness to help only when helping costs were high, indicating a trade-off between other-centric and egocentric motivations. These findings reveal that although people tend to be unwilling to help others pursue a luxury life, the motivations guiding this reluctance depend on individuals’ levels of agreeableness.

Altruistic behaviours are defined as “those behaviours intended to benefit another but which appear to have a high cost to the actor with little possibility of material or social reward” (Bryan & London, 1970, p. 200). Helping behaviour is a typical type of altruistic behaviour. Previous studies have differentiated high and low need (Sierksma et al., 2014; Wagner & Wheeler, 1969), the severity of need (Batson et al., 1986, 1988, 1991), and the degree of need (Bekkers & Wiepking, 2011) to investigate the effect of need on helping. These studies mainly examine peoples’ willingness to help others maintain a basic life. People’s willingness to help others pursue a luxury life and the motivations behind it have rarely been studied, although the demand for a luxury life exists. Hao et al. (2023) differentiated the demand for necessary items such as warm clothes from the demand for luxury items such as expensive entertainment products when investigating helping. Necessary items are life requisites that relieve discomfort, whereas luxury items bring abundance and pleasure (Berry, 1994). The demand for luxury items is thus essentially different from the demand for necessary items. Hao et al. (2023) found that the demand for luxury items reduced willingness to help, but it is not clear whether the finding is stable across situations and individuals. Moreover, the motivations underlying such unwillingness remain unclear.

## 1. Demand for Necessary Versus Luxury Items and Willingness to Help Within a Personality × Situation Framework

Based on a personality × situation perspective on helping (Bereczkei et al., 2010; Graziano et al., 2007), situations and personalities need to be considered when investigating helping. In helping situations, why others experience specific adversity is usually key information for helpers. The attribution–affect model (Weiner, 1980) proposes that attribution, especially the dimension of controllability (Meyer & Mulherin, 1980), plays an important role in willingness to help or helping behaviour. When the cause of others’ adversity is perceived to be uncontrollable, people are sympathetic and willing to help; when the cause of others’ adversity is perceived to be controllable, people are angry and unwilling to help (Weiner, 1980). Empirical studies have confirmed this model (Mackay & Barrowclough, 2005; Reisenzein, 1986; Rudolph et al., 2004; Tscharaktschiew & Rudolph, 2016). Whether the demand for luxury items reduces willingness to help in situations of different attribution is not clear. Because the demand for luxury items reflects the pursuit of abundance and pleasure, it may reduce willingness to help regardless of whether others’ adversity is attributed to controllable or uncontrollable causes. However, unlike uncontrollable causes, when others’ adversity is attributed to controllable causes, people are angry and may be insensitive to others’ demand. Thus, the effect of the demand for necessary versus luxury items on willingness to help is likely to be weaker under controllable attribution. Therefore, it was hypothesised that the effect of the demand for necessary versus luxury items on willingness to help is affected by attribution.

With respect to personality factors, previous studies have shown that agreeableness is especially related to altruistic behaviour. Agreeableness represents individual differences in motivation to maintain positive interpersonal relationships (Graziano & Tobin, 2009, 2013). Individuals with high agreeableness are always regarded as warm, friendly, and kind (Schmitt et al., 2007). Some studies did not find associations between agreeableness and helping behaviour (e.g., Lefevor & Fowers, 2016), but more studies have confirmed this association, indicating that individuals with high agreeableness have a stronger prosocial orientation and are more willing to help a wide range of victims or others in need of help (Graziano et al., 2007; Habashi et al., 2016; Hilbig et al., 2014). As mentioned above, the demand for luxury items may reduce willingness to help to a lesser extent under controllable attribution than under uncontrollable attribution. Whether the moderating effect exists in individuals with different levels of agreeableness should be further clarified. Individuals with high agreeableness care about interpersonal relationships and others’ welfare. They may understand others’ adversity due to controllable causes as well as that due to uncontrollable causes and be prepared to provide help under both controllable and uncontrollable attribution. Thus, the effect of the demand for necessary versus luxury items on willingness to help is likely to be similar under controllable and uncontrollable attribution in individuals with high agreeableness. By contrast, individuals with low agreeableness are less likely to understand others’ adversity due to controllable causes, and may be angry and insensitive to others’ demand under controllable attribution. For these individuals, the effect of the demand for necessary versus luxury items on willingness to help is likely to be smaller under controllable attribution. Therefore, it was hypothesised that the effect of the demand for necessary versus luxury items on willingness to help is moderated by attribution and the moderating effect is moderated by agreeableness of helpers.

## 2. Cost, Utilitarian Goals, Agreeableness of Helpers and Willingness to Help

More importantly, the reasons for unwillingness to help others pursue a luxury life remain unclear. The essential differences between necessary and luxury items lie in their price and utilitarian goals (Azgad-Tromer, 2015; Wiedmann et al., 2009). Necessary items are low-priced items with high utilitarian goals, whereas luxury items are high-priced items with low utilitarian goals. Clarifying whether unwillingness to help others obtain luxury items is due to high price or low utilitarian goals helps to reveal motivations behind helping decision-making. Haski-Leventhal (2009) summarised the debate over two opposite motivations for altruistic behaviour. Egocentric motivations emphasize that people display altruistic behaviour for their own interests because they are rational and economic beings. Other-centric motivations emphasize that people display altruistic behaviour to benefit others because of their adherence to the core values of humanity (Haski-Leventhal, 2009). Similarly, based on Batson’s (2011) work, Eisenberg et al. (2016) summarised motivations for prosocial behaviour from egoistic to altruistic motivations.

According to these theories, if the price of what others want affects willingness to help, it means that people may focus on their own cost of helping, suggesting egocentric motivations for helping. If the utilitarian goals of what others want affect willingness to help, it means that people may focus on the practicality of these items to others, suggesting other-centric motivations for helping. Previous studies have shown that willingness to help or helping behaviour is lower when the cost of helping is greater (Bell et al., 1995; Dovidio, 1982; Fritzsche et al., 2000; Iyer & Kanekar, 1991; Schneider, 1977) but have not systematically examined the effects of cost and utilitarian goals on willingness to help. Scholars believe that egoistic and altruistic motivations for prosocial behaviour can exist simultaneously (Batson, 2011; Eisenberg et al., 2016). Thus, it was hypothesised that both price and utilitarian goals affect willingness to help.

Moreover, it is also not clear whether price and utilitarian goals affect willingness to help in individuals with different levels of agreeableness. As mentioned above, from the perspective of other-centric motivations, utilitarian goals may affect willingness to help. Specifically, low utilitarian goals may reduce willingness to help. However, this may be the case only when others want high-priced items. When others want low-priced items, the cost of helping is relatively small. Individuals, especially those who are kind, are likely to ignore the utilitarian goals of what others want and try to help. Thus, the effect of utilitarian goals on willingness to help may be moderated by price. Individuals with high agreeableness are more willing to maintain positive relationships with others than individuals with low agreeableness (Gleason et al., 2004), because compassion, described as an other-oriented feeling, is an important aspect of agreeableness (Crowe et al., 2018). Accordingly, individuals with high agreeableness may have other-centric motivations for helping. The moderating effect is likely to be stronger in individuals with high agreeableness. Therefore, it was hypothesised that the effect of utilitarian goals on willingness to help is moderated by price and the moderating effect is moderated by agreeableness. From the perspective of egocentric motivations, price may affect willingness to help. Specifically, high price may reduce willingness to help. High price always means high cost of helping, and thus may reduce willingness to help regardless of utilitarian goals. Individuals with low agreeableness, as less warm, friendly and kind persons, may have egocentric motivations for helping. The effect of price on willingness to help is likely to be stronger in individuals with low agreeableness. Thus, it was hypothesised that the effect of price on willingness to help is moderated by agreeableness.

## 3. The Present Study

The present study aimed to investigate people’s willingness to help others pursue a luxury life and the motivations behind it. Study 1 examined whether the demand for luxury items reduced willingness to help, taking agreeableness of helpers and situations relating to controllability of causes of others’ adversity into account. Because the essential differences between necessary and luxury items lie in price and utilitarian goals, Study 2 further examined whether price or utilitarian goals affected willingness to help in individuals with different levels of agreeableness.

In each study, a common helping situation, lending money (Meyer & Mulherin, 1980), was presented to different experimental groups via different hypothetical scenarios. The participants were asked to report their willingness to help. In addition, emotion expectancy, namely, the emotions that people anticipate when evaluating possible behaviour, are related to their behavioural decision-making (Tangney et al., 2007). People’s expected emotions from helping reflects their willingness to help to some extent. Thus, the participants were also asked to report their emotion expectancy. They needed to answer how they would feel if they chose to help. With regard to agreeableness, the NEO Five-Factor Inventory (Costa & McCrae, 1992) was used to measure this personality trait. 

## 4. Study 1

### 4.1. Method

#### 4.1.1. Participants

A total of 156 participants (84 females and 72 males) were recruited from a university in Beijing, China. Their ages ranged from 17 to 36 years (*M* = 22.85, *SD* = 2.65). They were undergraduates or graduate students. The experiment was approved by the Psychological Ethics Committee of Capital Normal University.

#### 4.1.2. Experimental Design

The experiment adopted a 2 (demand) × 2 (attribution) between-subjects design. The participants were randomly assigned to one of four groups and read different helping scenarios in which a person wanted to borrow money to buy an item. The scenarios differed in whether the person wanted a necessary or luxury item and whether the person’s demand was due to uncontrollable or controllable causes.

#### 4.1.3. Experimental Material

The experimental materials were adapted from Hao et al. (2023). With respect to demand, as defined by Azgad-Tromer (2015) and Wiedmann et al. (2009), necessary items are low-priced items with high utilitarian goals, whereas luxury items are high-priced items with low utilitarian goals. In the present helping scenarios, the person wanted to borrow money to buy either a necessary item (e.g., a warm clothing) or a luxury item (e.g., an expensive game console). These items were selected for several reasons. First, an ordinary piece of warm clothing is a low-priced item with a high utilitarian goal (to keep warm) and thus is a necessary item. An expensive game console is a high-priced item with a low utilitarian goal (for pleasure) and thus is a luxury item. Second, the same kind of items may have the same utilitarian goals without specific explanations, so different kind of items were used to exclude this interference. Third, items such as clothing and game consoles are common objects in university students’ lives. With respect to attribution, in the present helping scenarios, the person was short of money because of either an uncontrollable cause (e.g., family members’ illness) or a controllable cause (e.g., buying lots of gaming equipment). At the end of each scenario, the participants were told “you just have some spare money” to exclude any interference caused by uncertainty about their helping ability. The sample *uncontrollable attribution–luxury item* scenario is as follows:

*Wang Ming is one of your classmates. Recently, his father was ill, and his life is very difficult. He wants to buy an expensive game console, but he does not have enough money. He finds you and asks whether you can lend him some money. You just have some spare money.*

#### 4.1.4. Measures and Procedure

After reading the scenarios, the participants were asked to rate their willingness to help and emotion expectancy. The participants were asked how willing they were to help the person by responding on a six-point scale from 1 (very unwilling) to 6 (very willing) and how they would feel if they lent the money by responding on a six-point scale from 1 (very unhappy) to 6 (very happy). Each group read two scenarios to increase the reliability of the measurement. The willingness-to-help scores in the two scenarios were significantly correlated (*p* < 0.001). The emotion expectancy scores in the two scenarios were also significantly correlated (*p* < 0.001). Thus, the average scores of the two scenarios were calculated and used for analysis.

A Chinese version of the NEO Five-Factor Inventory (Costa & McCrae, 1992) was used to assess the Big Five personality traits, but only agreeableness scores were analyzed. Each facet has 12 items. The participants were asked to evaluate how relevant each item was to themselves by responding on a five-point scale from 1 (strongly disagree) to 5 (strongly agree). An average score of the 12 items of agreeableness was calculated. In the Chinese version, the internal consistency reliabilities (*α*) of the neuroticism, extraversion, openness, agreeableness and conscientiousness facets are 0.77, 0.78, 0.63, 0.72 and 0.74, respectively (Yao & Liang, 2010). In the study, the internal consistency reliabilities (*α*) of the neuroticism, extraversion, openness, agreeableness and conscientiousness facets were 0.82, 0.76, 0.73, 0.64, and 0.80, respectively. Informed consent was obtained from each participant.

#### 4.1.5. Statistical Analysis

SPSS 26.0 was used for data analysis. ANOVAs (Fisher, 1925) and bootstrapping analysis (Efron, 1979) were conducted. First, 2 (demand) × 2 (attribution) ANOVAs were conducted to examine whether the effects of the demand for necessary versus luxury items on willingness to help and emotion expectancy were affected by attribution. Second, bootstrapping analysis was conducted with Model 3 of the PROCESS macro (Hayes, 2013). This analysis was used to examine whether the effects of the demand for necessary versus luxury items on willingness to help and emotion expectancy were moderated by attribution and whether the moderating effects were moderated by agreeableness. The number of bootstrap samples was set at 1000, and 95% confidence intervals (CIs) were used to estimate the significance of the moderating effects. Agreeableness scores were centred on means before analysis. The effects of the demand for necessary versus luxury items on willingness to help and emotion expectancy were estimated under different attribution (uncontrollable and controllable attribution) and different levels of agreeableness (one standard deviation above and below the mean).

### 4.2. Results and Discussion

**Figure 1:**
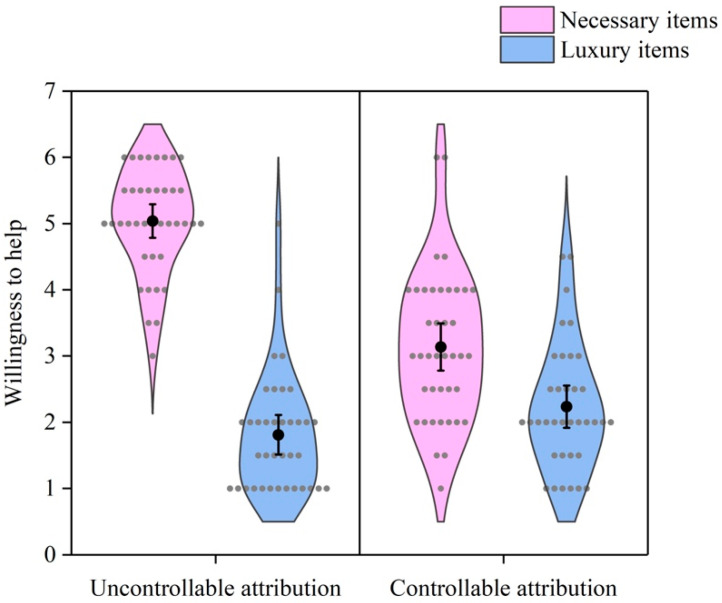
Willingness to help by demand and attribution. Gray dots represent individual data points. Black dots represent means. Error bars represent 95% confidence intervals.

As Figure 1 shows, a 2 (demand) × 2 (attribution) ANOVA revealed a significant interaction effect of demand and attribution on willingness to help, *F* (1, 152) = 58.19, *p* < 0.001, *η*^2^ = 0.277. Simple effect analysis revealed that the participants were less willing to help others obtain luxury items than necessary items under uncontrollable attribution, *t* = 16.72, *p* < 0.001. The effect of demand on willingness to help was weaker under controllable attribution, *t* = 3.59, *p* = 0.001. In addition, the main effects of demand and attribution were significant, *F* (1, 152) = 174.01, *p* < 0.001, *η*^2^ = 0.534; *F* (1, 152) = 21.47, *p* < 0.001, *η*^2^ = 0.124.

**Figure 2:**
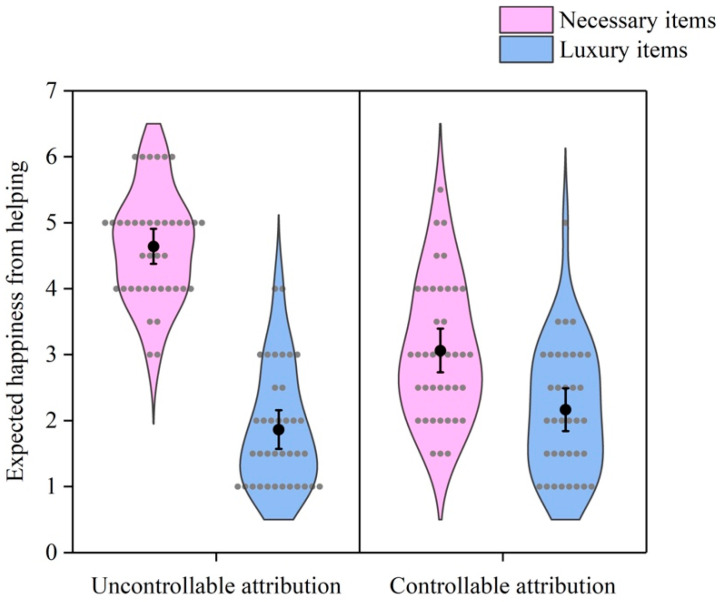
Emotion expectancy by demand and attribution. Gray dots represent individual data points. Black dots represent means. Error bars represent 95% confidence intervals.

As Figure 2 shows, a 2 (demand) × 2 (attribution) ANOVA also revealed a significant interaction effect of demand and attribution on emotion expectancy, *F* (1, 152) = 40.73, *p* < 0.001, *η*^2^ = 0.211. Simple effect analysis revealed that the participants expected less happiness from helping others obtain luxury items than from helping others obtain necessary items under uncontrollable attribution, *t* = 14.25, *p* < 0.001. The effect of demand on emotion expectancy was weaker under controllable attribution, *t* = 4.01, *p* < 0.001. In addition, the main effects of demand and attribution on emotion expectancy were significant, *F* (1, 152) = 153.25, *p* < 0.001, *η*^2^ = 0.502; *F* (1, 152) = 18.37, *p* < 0.001, *η*^2^ = 0.108. These results indicated that the demand for luxury items reduced willingness to help and expected happiness from helping, but the effect was smaller under controllable attribution than under uncontrollable attribution.

**Figure 3:**
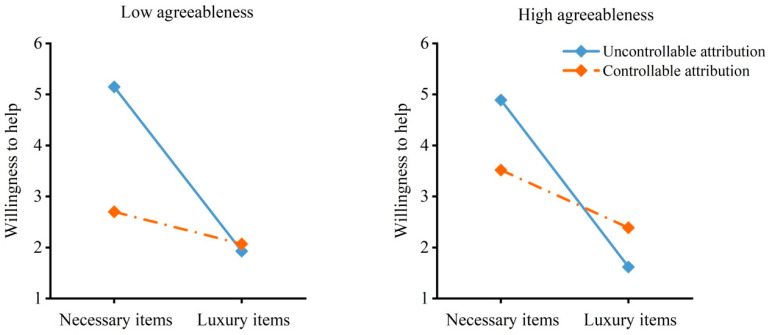
Willingness to help by demand, attribution and agreeableness. The moderated moderation model indicated an interaction effect of demand and attribution on willingness to help.

As shown in Table 1, in the model with willingness to help as the dependent variable, the three-way interaction effect of demand, attribution and agreeableness was not significant, but the two-way interaction effect of demand and attribution was significant. Specifically, as shown in Figure 3, individuals with low agreeableness were less willing to help others obtain luxury items than necessary items under uncontrollable attribution, *B* = −3.21, *SE* = 0.30, *t* = −10.81, *p* < 0.001, 95% CI = [−3.80, −2.62]; the effect was weaker under controllable attribution, *B* = −0.63, *SE* = 0.32, *t* = −1.97, *p* = 0.051, 95% CI = [−1.26, 0.002]. For individuals with high agreeableness, the results were similar. Individuals with high agreeableness were less willing to help others obtain luxury items than necessary items under uncontrollable attribution, *B* = −3.27, *SE* = 0.37, *t* = −8.87, *p* < 0.001, 95% CI = [−4.00, −2.54]. The effect was weaker under controllable attribution, *B* = −1.13, *SE* = 0.27, *t* = −4.17, *p* = 0.001, 95% CI = [−1.67, −0.60].

**Figure 4:**
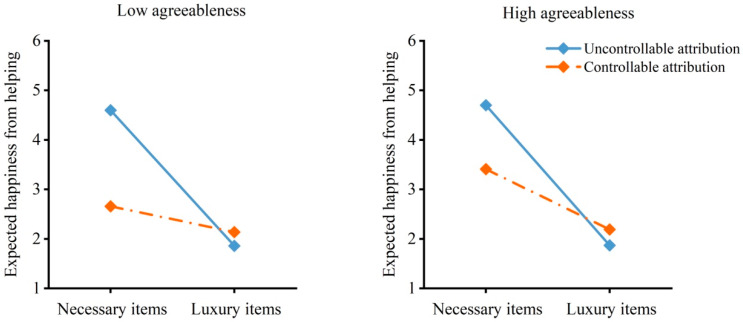
Emotion expectancy by demand, attribution and agreeableness. The moderated moderation model indicated an interaction effect of demand and attribution on emotion expectancy.

As shown in Table 1, in the model with emotion expectancy as the dependent variable, the three-way interaction effect of demand, attribution and agreeableness was not significant, but the two-way interaction effect of demand and attribution was significant. Specifically, as shown in Figure 4, for individuals with low agreeableness, they expected less happiness from helping others obtain luxury items than from helping others obtain necessary items under uncontrollable attribution, *B* = −2.74, *SE* = 0.29, *t* = −9.55, *p* < 0.001, 95% CI = [−3.30, −2.17]. The effect was weaker under controllable attribution, *B* = −0.52, *SE* = 0.31, *t* = −1.69, *p* = 0.093, 95% CI = [−1.13, 0.09]. For individuals with high agreeableness, the results were similar. Individuals with high agreeableness expected less happiness from helping others obtain luxury items than from helping others obtain necessary items under uncontrollable attribution, *B* = −2.83, *SE* = 0.36, *t* = −7.95, *p* < 0.001, 95% CI = [−3.53, −2.13]. The effect was weaker under controllable attribution, *B* = −1.22, *SE* = 0.26, *t* = −4.64, *p* < 0.001, 95% CI = [−1.74, −0.70]. These results indicated that the effects of the demand for necessary versus luxury items on willingness to help and emotion expectancy were moderated by attribution, but the moderating effects were not moderated by agreeableness. In other words, the demand for luxury items reduced willingness to help and expected happiness from helping to a lesser extent under controllable attribution than under uncontrollable attribution regardless of agreeableness.

Study 1 confirmed that the demand for luxury items reduced willingness to help and expected happiness from helping. Because the essential differences between necessary and luxury items lie in price and utilitarian goals, Study 2 aimed to further examine whether price or utilitarian goals affected willingness to help and emotion expectancy in individuals with different levels of agreeableness.

## 5. Study 2

### 5.1. Method

#### 5.1.1. Participants

A total of 152 participants (85 females and 67 males) were recruited from a university in Beijing, China. Their ages ranged from 18 to 30 years (*M* = 22.15, *SD* = 2.40). They were undergraduates or graduate students. The experiment was approved by the Psychological Ethics Committee of Capital Normal University.

#### 5.1.2. Experimental Design

The experiment adopted a 2 (price) × 2 (utilitarian goals) between-subjects design. The participants were randomly assigned to one of four groups and read different helping scenarios in which a person wanted to borrow money to buy an item. The scenarios differed in whether the person wanted a low-priced or high-priced item and whether the item had a low utilitarian goal or a high utilitarian goal.

#### 5.1.3. Experimental Materials

The experimental materials were adapted from Hao et al. (2023). Regarding price, in the present helping scenarios, the person wanted either a low-priced item (e.g., a cell phone case) or a high-priced item (e.g., a piece of expensive clothing). These items were selected for several reasons. First, an ordinary cell phone case is a low-priced item, and an expensive piece of clothing is a high-priced item. Second, expensive items may have not only higher price but also better quality than the same kind of ordinary items, so different items were used to exclude this interference. Third, items such as cell phone cases and clothing are common objects in university students’ lives. With respect to utilitarian goals, in the present helping scenarios, the item either had a low utilitarian goal (e.g., for abundance or pleasure) or a high utilitarian goal (e.g., for practical use). The sample *high price–high utilitarian goal* scenario is as follows:

*Li Hua is one of your classmates. He is having an important interview next week. He wants to buy a piece of expensive clothing to improve his image. He does not have enough money. He finds you and asks whether you can lend him some money. You just have some spare money.*

#### 5.1.4. Measures and Procedure

The measurement of willingness to help and emotion expectancy was similar to that used in Study 1. Each group read two scenarios to increase the reliability of the measurement. The willingness-to-help scores in the two scenarios were significantly correlated (*p* < 0.001). The emotion expectancy scores in the two scenarios were also significantly correlated (*p* < 0.001). Thus, the average scores of the two scenarios were calculated and used for analysis. The same Chinese version of the NEO Five-Factor Inventory (Costa & McCrae, 1992) was used to measure the Big Five personality traits, but only agreeableness scores were analyzed. In the study, the internal consistency reliabilities (*α*) of the neuroticism, extraversion, openness, agreeableness and conscientiousness facets were 0.86, 0.77, 0.71, 0.62, and 0.81, respectively. Informed consent was obtained from each participant.

#### 5.1.5. Statistical Analysis

SPSS 26.0 was used for data analysis. ANOVAs (Fisher, 1925) and bootstrapping analysis (Efron, 1979) were conducted. First, 2 (price) × 2 (utilitarian goals) ANOVAs were conducted to examine whether high price or low utilitarian goals reduced willingness to help and expected happiness from helping. Second, bootstrapping analysis was conducted with Model 3 of the PROCESS macro (Hayes, 2013). This analysis was used to examine whether the effects of utilitarian goals on willingness to help and emotion expectancy were moderated by price and whether the moderating effects were moderated by agreeableness. The number of bootstrap samples was set at 1000, and 95% confidence intervals (CIs) were used to estimate the significance of the moderating effects. Before bootstrapping analysis, agreeableness scores were centred on means. The effects of utilitarian goals on willingness to help and emotion expectancy were estimated at different price (low and high price) and levels of agreeableness (one standard deviation above and below the mean). Because the ANOVAs did not find the effects of price on willingness to help and emotion expectancy, whether the effects of price on willingness to help and emotion expectancy were moderated by agreeableness was not examined.

### 5.2. Results and Discussion

**Figure 5:**
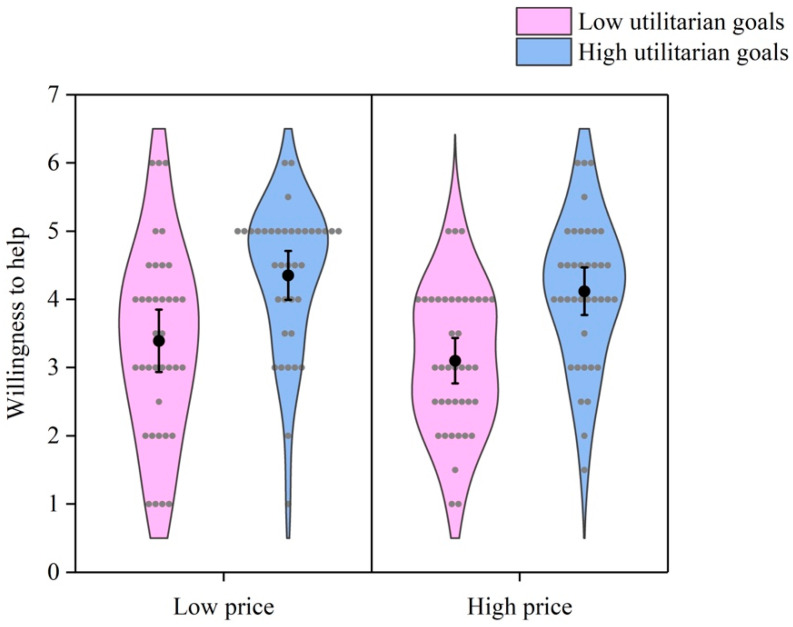
Willingness to help by price and utilitarian goals. Gray dots represent individual data points. Black dots represent means. Error bars represent 95% confidence intervals.

As Figure 5 shows, a 2 (price) × 2 (utilitarian goals) ANOVA did not find an interaction effect of price and utilitarian goals on willingness to help, *F* (1, 148) = 0.03, *p* = 0.874, *η*^2^ < 0.001. The main effect of price on willingness to help was not significant, *F* (1, 148) = 2.00, *p* = 0.159, *η*^2^ = 0.013. Nevertheless, the main effect of utilitarian goals was significant, *F* (1, 148) = 28.42, *p* < 0.001, *η*^2^ = 0.161. The participants were less willing to help others obtain items with low utilitarian goals than those with high utilitarian goals.

**Figure 6:**
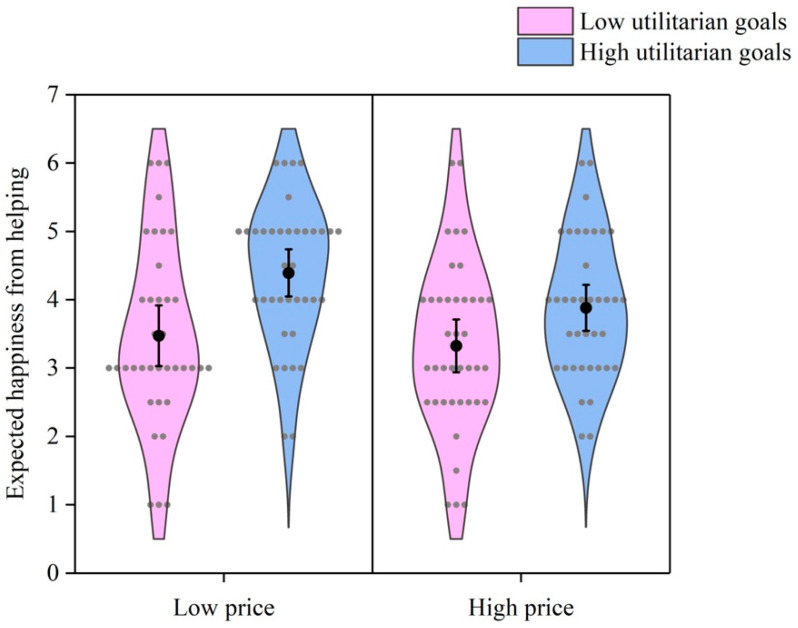
Emotion expectancy by price and utilitarian goals. Gray dots represent individual data points. Black dots represent means. Error bars represent 95% confidence intervals.

As Figure 6 shows, a 2 (price) × 2 (utilitarian goals) ANOVA revealed similar results, with emotion expectancy as the dependent variable. The interaction effect of the two factors was not significant, *F* (1, 148) = 0.93, *p* = 0.336, *η*^2^ = 0.006. The main effect of price was not significant, *F* (1, 148) = 3.07, *p* = 0.082, *η*^2^ = 0.020. It indicated that the participants expected similar levels of happiness from helping when others wanted low-priced or high-priced items. However, the main effect of utilitarian goals was significant, *F* (1, 148) = 15.44, *p* < 0.001, *η*^2^ = 0.094. It meant that the participants expected less happiness from helping others obtain useless items than from helping others obtain useful items. These results indicated that utilitarian goals, rather than price, affected willingness to help and emotion expectancy. Thus, whether the effects of utilitarian goals on willingness to help and emotion expectancy were moderated by price and whether the moderating effects were moderated by agreeableness were further investigated.

**Figure 7:**
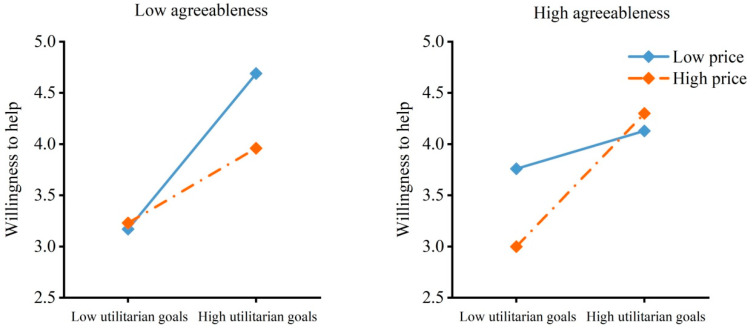
Willingness to help by utilitarian goals, price and agreeableness. The moderated moderation model indicated an interaction effect of utilitarian goals, price and agreeableness on willingness to help.

As shown in Table 2, the model with willingness to help as the dependent variable revealed a three-way interaction effect of utilitarian goals, price and agreeableness. Specifically, as shown in Figure 7, individuals with low agreeableness were less willing to help others obtain items with low utilitarian goals than those with high utilitarian goals when the price of the items was low, *B* = −1.52, *SE* = 0.43, *t* = −3.52, *p* < 0.001, 95% CI = [−2.37, −0.67]. The results were similar when the price of the items was high, *B* = −0.73, *SE* = 0.35, *t* = −2.07, *p* = 0.040, 95% CI = [−1.43, −0.03]. However, individuals with high agreeableness were willing to help others obtain items with low utilitarian goals as well as those with high utilitarian goals when the price of the items was low, *B* = −0.37, *SE* = 0.42, *t* = −0.88, *p* = 0.380, 95% CI = [−1.19, 0.46]. Items with low utilitarian goals reduced their willingness to help when the price of the items was high, *B* = −1.30, *SE* = 0.35, *t* = −3.76, *p* < 0.001, 95% CI = [−1.98, −0.62]. 

**Figure 8:**
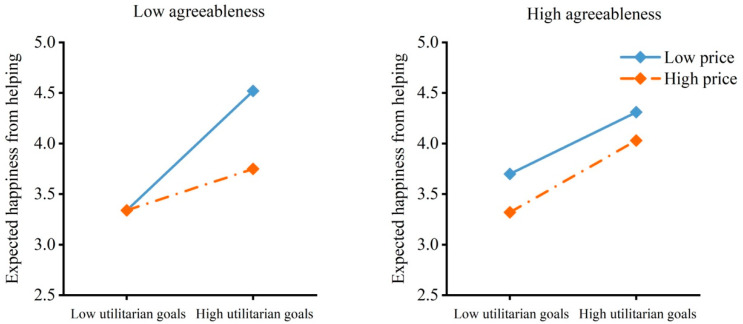
Emotion expectancy by utilitarian goals, price and agreeableness. The moderated moderation model indicated an effect of utilitarian goals on emotion expectancy.

As shown in Table 2, the model with emotion expectancy as the dependent variable revealed only an effect of utilitarian goals. As Figure 8 shows, the participants expected less happiness from helping others obtain items with low utilitarian goals than from helping others obtain those with high utilitarian goals regardless of the price of the items and their agreeableness. These results indicated that the effect of utilitarian goals on willingness to help was moderated by price and the moderating effect was moderated by agreeableness. In other words, for individuals with low agreeableness, low utilitarian goals reduced their willingness to help regardless of the cost of helping. For individuals with high agreeableness, low utilitarian goals reduced their willingness to help only when the cost of helping was high. In addition, low utilitarian goals reduced expected happiness from helping.

## 6. General Discussion

The present study aimed to reveal people’s willingness to help others pursue a luxury life and the motivations behind it. Study 1 revealed that the participants showed less willingness to help others obtain luxury items and expected less happiness from helping others obtain luxury items. Study 2 further focused on two important aspects that differentiated necessary and luxury items: price and utilitarian goals. The results indicated that utilitarian goals affected willingness to help and emotion expectancy. Moreover, for individuals with low agreeableness, low utilitarian goals reduced willingness to help regardless of the cost of helping; however, for individuals with high agreeableness, low utilitarian goals reduced willingness to help only when the cost of helping was high. In addition, low utilitarian goals reduced expected happiness from helping independent of the cost of helping and helpers’ agreeableness.

On the whole, Study 1 found that the participants were less willing to respond to the demand for luxury items than the demand for necessary items, which replicated Hao et al.’s (2023) findings. Previous studies have focused on various types of needs but have yielded inconsistent results. Wagner and Wheeler (1969) did not find an effect of high vs. low need on helping, but Bekkers and Wiepking (2011) concluded that the degree of need affected helping. These inconsistent results are yielded probably because demand is not essentially differentiated. The present study overcomes the shortcoming by differentiating the demand for necessary items and that for luxury items. On the basis of Maslow’s hierarchy of needs, Azgad-Tromer (2015) claimed that necessary items are obtained to satisfy basic need, whereas luxury items are obtained to satisfy higher levels of need. Thus, the participants were unwilling to help others obtain luxury items.

Consistent with the hypotheses, it was found that the effect of the demand for necessary versus luxury items on willingness to help was affected by attribution. Specifically, the demand for luxury items reduced willingness to help to a lesser extent under controllable attribution than under uncontrollable attribution. When the cause of another’s adversity is perceived to be controllable, people will feel angry (Rudolph et al., 2004). This negative emotion might make people insensitive to others’ demand; thus, the effect of demand on willingness to help weakens. Contrary to the hypotheses, the moderating effect of attribution on the relationship between the demand and willingness to help was not moderated by helpers’ agreeableness. In other words, when the cause of another’s adversity was perceived to be controllable, the effect of demand on willingness to help weakened regardless of helpers’ agreeableness. Under controllable attribution, individuals with both low and high agreeableness might experience anger, which made them insensitive to others’ demand.

Study 2 further clarified the motivations for unwillingness to respond to the demand for luxury items. The result that the participants’ willingness to help was constrained by low utilitarian goals rather than high price partially supported the hypotheses. According to theories of altruism in developmental psychology, individuals learn to control their impulses, namely, egoistic tendencies, only through time and socialisation (Haski-Leventhal, 2009). In other words, as individuals enter adulthood, continuous socialisation may make them care more about others’ welfare. Thus, in the present study, the participants, as young adults, showed other-centric motivations for helping, caring more about the utilitarian goals of what others wanted.

Consistent with the hypotheses, the effect of utilitarian goals on willingness to help was moderated by price, and the moderating effect was moderated by agreeableness. As expected, for individuals with high agreeableness, low utilitarian goals reduced willingness to help only when the price of the items was high. In other words, individuals with high agreeableness were unwilling to help when the items not only had low utilitarian goals for others but also had high cost of helping for themselves. This finding suggests that individuals with high agreeableness seem to weigh the interests of others and themselves in helping decision-making. These agreeable individuals’ cautious refusal to help reflects their motivation to maintain positive interpersonal relationships (Graziano & Tobin, 2009, 2013) and their prosociality, as previous studies have demonstrated. Pinazo et al. (2016) reported that agreeableness was associated with the activation of the precuneus, which is responsible for mentalizing. Mentalizing is closely related to prosocial behaviour (Majdandžić et al., 2016; Tamnes et al., 2018). Hilbig et al. (2014) reported that agreeableness is associated with prosocial orientation through the honesty–humility aspect, and Zhao et al. (2016) reported that the politeness aspect of agreeableness is associated with prosociality. Unexpectedly, low utilitarian goals also reduced willingness to help in individuals with low agreeableness. This result suggests that individuals with low agreeableness may also have other-centric motivations in helping decision-making. Different from agreeable individuals, individuals with low agreeableness do not have much motivation to maintain positive interpersonal relationships. Therefore, in the present study, they were unwilling to help others obtain useless items even if the cost of helping was small.

In addition to willingness to help, emotion expectancy was also measured. Emotion expectancy or emotion attribution is found to be associated with prosocial or antisocial behaviour (Johnston & Krettenauer, 2011; Krettenauer & Eichler, 2006; Malti & Krettenauer, 2013). Willingness to help is also related to helping behaviour to some extent. Thus, the results related to emotion expectancy replicated those related to willingness to help in Study 1. However, in Study 2, a moderated moderating effect was found for willingness to help rather than emotion expectancy. Only utilitarian goals affected emotion expectancy. Emotion is a faster and automatic response to stimuli. Expected unhappiness from helping may be evoked at once by others’ demand for items with low utilitarian goals.

Despite the above important findings, the present study has some limitations. First, the effects of demand, attribution, utilitarian goals and price on willingness to help and emotion expectancy were examined through hypothetical scenarios due to feasibility. Future studies need to assess actual behaviour to further confirm the present findings. Second, the present study examined people’s willingness to help others pursue a luxury life and the motivations for it only in individuals with different levels of agreeableness. For individuals with different levels of neuroticism, extraversion, openness or conscientiousness, their responses to others’ pursuit of a luxury life and the motivations for it are not clear and need to be clarified in future studies.

In conclusion, the present study reveals that although people tend to be unwilling to help others pursue a luxury life, the motivations guiding this reluctance depend on individuals’ levels of agreeableness.

## Figures and Tables

**Table 1:** Bootstrapping analysis predicting willingness to help and emotion expectancy by demand, attribution and agreeableness.

		*R* ^2^	*F*	*p*	Δ*R*^2^	*F*	*p*	*B*	*SE*	*t*	*p*	95% CI
Willingness to help	Demand	0.65	38.71	<0.001	0.00	0.48	0.491	−5.60	0.50	−11.30	<0.001	[−6.58, −4.62]
	Attribution							−4.27	0.49	−8.78	<0.001	[−5.23, −3.31]
	Agreeableness							−1.88	1.92	−0.98	0.329	[−5.66, 1.91]
	Demand × Attribution							2.36	0.31	7.60	<0.001	[1.75, 2.98]
	Demand × Agreeableness							0.42	1.18	0.35	0.724	[−1.91, 2.75]
	Attribution × Agreeableness							1.66	1.15	1.45	0.150	[−0.61, 3.93]
	Demand × Attribution × Agreeableness							−0.49	0.70	−0.69	0.491	[−1.87, 0.90]
Emotion expectancy	Demand	0.60	31.50	<0.001	0.00	0.95	0.331	−4.70	0.48	−9.82	<0.001	[−5.64, −3.75]
	Attribution							−3.53	0.47	−7.52	<0.001	[−4.46, −2.60]
	Agreeableness							−1.14	1.85	−0.62	0.538	[−4.80, 2.51]
	Demand × Attribution							1.92	0.30	6.39	<0.001	[1.32, 2.51]
	Demand × Agreeableness							0.56	1.14	0.49	0.624	[−1.69, 2.81]
	Attribution × Agreeableness							1.36	1.11	1.23	0.221	[−0.83, 3.55]
	Demand × Attribution × Agreeableness							−0.66	0.68	−0.97	0.331	[−2.00, 0.68]

*Note:* B = unstandardized coefficient. CI = confidence interval.

**Table 2:** Bootstrapping analysis predicting willingness to help and emotion expectancy by utilitarian goals, price and agreeableness.

		*R* ^2^	*F*	*p*	Δ*R*^2^	*F*	*p*	*B*	*SE*	*t*	*p*	95% CI
Willingness to help	Utilitarian goals	0.20	5.13	<0.001	0.03	4.61	0.034	−0.87	0.61	−1.43	0.154	[−2.06, 0.33]
	Price							−0.20	0.60	−0.34	0.737	[−1.38, 0.98]
	Agreeableness							−4.83	2.58	−1.87	0.064	[−9.93, 0.28]
	Utilitarian goals × Price							−0.08	0.38	−0.20	0.843	[−0.82, 0.67]
	Utilitarian goals × Agreeableness							3.20	1.54	2.08	0.039	[0.16, 6.24]
	Price × Agreeableness							2.92	1.47	1.98	0.049	[0.01, 5.83]
	Utilitarian goals × Price × Agreeableness							−1.92	0.89	−2.15	0.034	[−3.68, −0.15]
Emotion expectancy	Utilitarian goals	0.13	2.98	0.006	0.01	1.15	0.285	−1.23	0.62	−1.98	0.050	[−2.45, −0.00]
	Price							−0.85	0.61	−1.39	0.166	[−2.06, 0.36]
	Agreeableness							−2.41	2.64	−0.91	0.363	[−7.63, 2.81]
	Utilitarian goals × Price							0.33	0.39	0.86	0.393	[−0.43, 1.09]
	Utilitarian goals × Agreeableness							1.62	1.57	1.03	0.304	[−1.49, 4.73]
	Price × Agreeableness							1.53	1.51	1.02	0.310	[−1.44, 4.51]
	Utilitarian goals × Price × Agreeableness							−0.98	0.91	−1.07	0.285	[−2.79, 0.83]

*Note:* B = unstandardized coefficient. CI = confidence interval.

## Data Availability

Data are available upon request from the corresponding author.
